# The effect of training schedule and playing positions on training loads and game demands in professional handball players

**DOI:** 10.5114/biolsport.2023.121323

**Published:** 2022-12-13

**Authors:** Roger Font Ribas, Claude Karcher, Eduard Loscos-Fàbregas, Albert Altarriba-Bartés, Javier Peña, Jordi Vicens-Bordas, Jose Antonio Mesas, Alfredo Irurtia

**Affiliations:** 1 Sport Performance Area FC Barcelona, Barcelona, Spain; 2 National Institute of Physical Education of Catalonia (INEFC), University of Barcelona (UB), Barcelona, Spain; 3 School of Health Sciences, Tecnocampus, Pompeu Fabra University, Mataró, Spain; 4 University of Strasbourg, Faculty of Medicine, Mitochondria, Oxidative Stress and Muscular Protection laboratory (EA 3072), Strasbourg, France; 5 University of Strasbourg, Faculty of Sport Sciences, European Centre for Education, Research and Innovation in Exercise Physiology (CEERIPE), Strasbourg, France; 6 Sport and Physical Activity Studies Centre (CEEAF), University of Vic- Central University of Catalonia (UVic-UCC), Barcelona, Spain; 7 Sport Performance Analysis Research Group (SPARG), University of Vic- Central University of Catalonia (UVic-UCC), Barcelona, Spain; 8 INEFC-Barcelona Sport Sciences Research Group, National Institute of Physical Education of Catalonia (INEFC), University of Barcelona (UB), Barcelona, Spain; 9 Catalan School of Kinanthropometry, National Institute of Physical Education of Catalonia (INEFC), University of Barcelona (UB), Barcelona, Spain

**Keywords:** External load, Team sports, IMUs, Internal load, RPE

## Abstract

In this research, we aimed to (1) describe the differences in internal and external load between playing positions and (2) characterize the training demands of the days before competitive events for professional handball players. Fifteen players (5 wings, 2 centre backs, 4 backs, and 2 pivots) were equipped with a local positioning system device during training and 11 official matches. External (total distance, high-speed running, player load) and internal loads (rating of perceived exertion) were computed. Substantial differences were recorded between the external load variables depending on each playing position and depending on whether it was a training day (high-speed running: effect size (ES) ≥ 2.07; player load: ES ≥ 1.89) or a match (total distance: ES ≥ 1.27; high-speed running: ES ≥ 1.42; player load: ES ≥ 1.33). Differences in internal load were not substantial. The rating of perceived exertion, at this competitive level, does not seem to discriminate the differences registered in the external load, probably due to the degree of adaptation to the specific effort of these players. The large differences observed in external load variables should be used to tailor practices and better adjust the training demands in professional handball settings.

## INTRODUCTION

In-season load monitoring is relevant in high-performance team sports as this process gives critical information to the technical staff. This information allows coaches to adapt the training contents, helping players perform to the best of their abilities during games [1] with reduced injury risk [2]. Despite this relevance, no studies, to the best of our knowledge, have analysed the training demands in handball as in other team sports such as football [3].

Previous studies in handball suggest that many factors affect the playing demands during games. Thus, gender (women travelled higher total distance than men) [4, 5], playing level (amateur handball players accumulated a lower total distance) [6], or age (adolescent handball players showed lower levels of exercise intensity, in the second half of matches) [7] have been reported in different pieces of research. It is worth noting that many studies [5, 8] show that playing positions modulate the game demands considerably because tactical roles attributed to each playing position are very specific [9, 10].

Thus, the external [5, 8, 11, 12] and internal load game demands [13, 14]. For instance, pivots (PIV) travel lower total distances (3149 ± 639 m) [5], while wings perform the highest number of sprints [8] and cover the highest high-speed running (HSR) distances (1229 ± 129.4 m) [11]. Playing position also influences internal load substantially [13, 14]. Povoas et al. [15] found that back players and pivots had the highest average heart rate (HR) values and total game time at intensities > 80% HRmax. Therefore, and considering these previous results, monitoring game demands is relevant. However, training sessions represent the most significant part of the weekly training load (at least in volume), and we should accurately monitor them. We hypothesize that training demands are influenced mainly by playing positions, and these differences could have substantial consequences in training load management [16] and could explain injury rates [2]. Understanding these variations may improve load management [16] and mitigate injury risks [2]. Optimizing training to prepare players for competition with specific tasks also seems essential [17].

It is also worth noting that physical profiles differ between playing positions [18, 19], also influencing training loads [20]. Thus, body dimensions are relevant as they can alter training load [21, 22]. These differences add disparity to the training response, making training individualization necessary [18, 19].

Another aspect to consider is the management of training loads to help players perform during games to the maximum of their abilities. Many studies have shown that periodization is crucial for performance [16] and injury prevention [2].

To be physically prepared for the match, players must train to develop specific physical skills (e.g., lower limb muscle power and ability to accelerate) in an optimal manner close to or superior to competitive demands [17]. The knowledge of such game demands can lead professionals to apply the approach “train as you play” [23]; however, despite its importance, there appears to be no study in handball that provides this information. We do not know, for example, whether games offer the highest load of the microcycle. It is paramount, then, to compare training and game demands. Training load management control is a crucial driver of performance in a team sport [16] and a strategic advantage for the different coaching staff [3].

The aims of this research are 1) to describe the internal and external training load differences between playing positions and 2) to characterize the training demands to compete for every training day. Therefore, we examined the differences in internal (RPE) and external (using inertial measurement units (IMUs)) loading concerning training days, playing positions, and competitive playing demands. Our findings should help coaches to design playing positions’ specific training content related to game demands.

## MATERIALS AND METHODS

### Experimental approach to the problem

We conducted a cross-sectional, observational study to determine the differences between playing positions during games and practices of a team playing in the second division of the Spanish handball competition during the 2018–19 season. The reported results consider the average values of 11 competitive home games and 25 weeks of practice. There were usually four training sessions a week; the day before the competition (match day; MD) was MD-1, two days before the competition (MD-2), and so on until MD-5. The research data emerged thanks to the daily monitoring of the players conducted in training and competition; therefore, relevant approval of the ethics committee was not required [24]. The study was conducted following the ethical principles for biomedical research with human beings, established in the Declaration of Helsinki of the World Medical Association (updated in 2013), and the club’s managerial structure approved its implementation.

### Subjects

Fifteen professional handball players participated in this study. Some of the players were internationals with their national teams as they were still in their formative stages. Players were grouped according to their usual playing position during the competition (Table 1).

**TABLE 1:** Physical characteristics of the players (mean ± standard deviation).

Position	Mean (n)	Age (years)	Body mass (kg)	Height (cm)
Left wings (LW)	3	23.0 ± 0.0	78.5 ± 3.5	176.0 ± 0.0
Right wings (RW)	2	23.5 ± 0.7	73.0 ± 2.8	179.0 ± 1.4
Centre backs (CB)	3	24.0 ± 1.0	90.3 ± 9.3	190.3 ± 7.5
Left back (LB)	3	23.7 ± 0.6	93.0 ± 6.6	192.3 ± 3.5
Right back (RB)	2	23.0 ± 0.0	89.5 ± 16.3	194.5 ± 9.2
Pivot (PIV)	2	29.5 ± 4.9	100.5 ± 7.8	192.5 ± 3.5

### Sessions and games monitoring

The study was carried out using the WIMU PRO system (RealTrack Systems SL, Almería, Spain). Each device, whose dimensions were 81 × 45 × 16 mm (height/width/depth) and which weighed 70 g, was fitted to the back of each player with an adjustable vest (Rasán, Valencia, Spain).

In training, the recording was uninterrupted. During games, playing time was only recorded when the players were on the court. The time spent between player rotation, team time outs (TTO) (a maximum of three per team), periods when the game was interrupted, and the disciplinary sanctions typical of handball, where players must leave the court for two minutes, were omitted.

Table 2 describes the different training days’ main characteristics (objectives, volume, duration, intensity, static or dynamic training).

**TABLE 2:** Description of the objectives, contents and orientation of the volume and intensity related to the training days.

MD	Aim	Conditional work	Static phase (%)	Full court game (%)	Volume	Intensity	Training session time (min)
MD -5	Individual development	Structural	80	20	***	**	87.9 ± 8.2
MD -4	Individual development	Structural	60	40	****	**	97.5 ± 15.7
MD -3	Tactical session	Structural	60	40	****	***	95.0 ± 11.4
MD -2	Match preparation	Optimising	50	50	***	***	90.1 ± 11.5
MD -1	Match preparation	Optimising	70	30	**	*****	86.4 ± 8.5

Note: MD: match day.

### Data processing

The positioning data record was monitored in real time and subsequently analysed using the SPRO software version 946–949 (SPRO, RealTrack Systems, 2018). The system operates using triangulations between four antennas with patented ultra-wideband technology (18 Hz sampling frequency) placed 5 m away from each one of the corners of the court and at the height of 6 m. These units include several sensors that record at different sampling frequencies. The sampling frequency used for 3-axis accelerometer, gyroscope, and magnetometer was 100 Hz and 120 kPa for the barometer [25, 26].

Total distance (TD, in metres) and high-speed running (HSR, distance covered in metres above 18.1 kph) [5, 7] were extracted from the root data reported by the system using SPRO software. The player load (PL; arbitrary units, au) was calculated as the square root of the sum of the squared instantaneous rates of change in acceleration in each of the three planes divided by 100 in absolute [27].

Internal load (IL) data were obtained through the RPE-based method (arbitrary units, au) [28, 29] at 10–30 minutes following every handball training session and game.

The recording of all training sessions and games resulted in 1033 individual records for the external load and 1008 files for the internal load.

### Statistical analysis

Data in the text and figures are presented as means with standard deviation (SD). All data were first log-transformed to avoid bias arising from non-uniformity errors. Standardized differences in the mean (Cohen’s effect size) were calculated to compare external and internal load between and within the different playing positions during games and training sessions. Effect size comparisons were rated using the Hopkins scale: 0.2 (small), 0.6 (moderate), 1.2 (large), 2.0 (very large) [30]. The 90% confidence interval was also calculated for each effect size. Considering the high number of comparisons, the results were not analysed when the lower limit of the effect size was below 0.6. The percentage of game demands for each variable was also calculated following this formula:

Statistical analyses were performed using RStudio (v1.3) and the Esvis package (v0.3.1).

## RESULTS

**FIG. 1:**
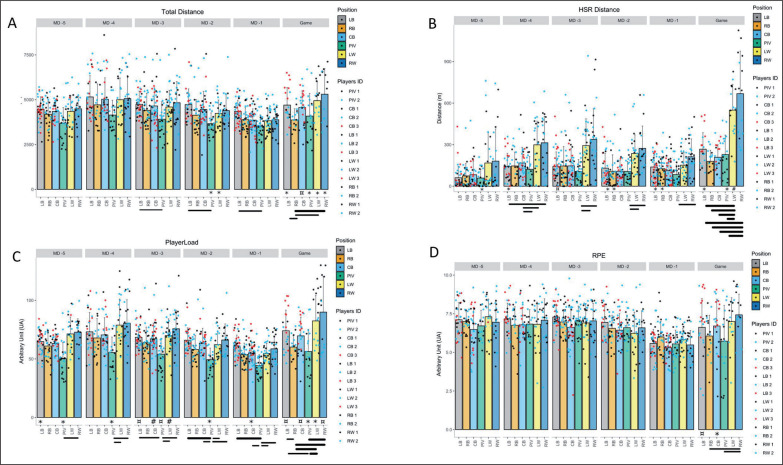
Comparison between playing position related to game day and total distance (A), high-speed running distance (B), player load (C), and rate of perceived exertion (D). Bar graphs represent the mean and standard deviation for each playing position related to game day. Coloured dots stand for individual value within the playing position and game day. The thickness of the lines represents the magnitude of the difference (effect size): ━ very large difference; ─ large difference; ― moderate difference. The magnitude of the difference of the within playing position is represented by ¤ when it is large very large, * when it is large and # when it is moderate. Only effect sizes with a lower limit above 0.6 are shown.

Table 3 summarizes each variable’s mean value and standard deviation for playing positions and game day. Figure 1 shows total distance (TD), high-speed running distance (HSR), player load (PL), and rating of perceived exertion for each day of training, for each playing position, and for each player.

**TABLE 3:** Comparison of the mean value of each indicator related to training day and games. Direction of the arrow describe the magnitude of the standardized difference, horizontal arrow stand for an effect size between 0.6 and -0.6, down arrow for an effect size above 1.2, diagonal down arrow for an effect size between -0.6 and -1.2 and diagonal up for an effect size between 0.6 and 1.2.

		CB	LB	LW	PIV	RB	RW
**Total distance (m)**	**Game**	**4562.7 ± 928.4**	**4699.5 ± 974.2**	**4946.1 ± 1051.0**	**4084.8 ± 1263.83**	**3872.0 ± 623.3**	**5306.6 ± 1422.2**
	**MD -1**	↘3861.9 ± 627.6	→4347.5 ± 623.6	↓3826.0 ± 654.7	→3526.4 ± 623.9	→3888.2 ± 663.9	↓3882.3 ± 628.3
	**MD -2**	→4457.0 ± 1008.2	→4731.6 ± 871.8	↘4236.9 ± 747.2	→3665.6 ± 597.7	→4136.1 ± 881.8	↘4394.7 ± 897.5
	**MD -3**	→4650.1 ± 1012.7	→4744.1 ± 730.9	→4593.2 ± 928.4	→3913.3 ± 969.7	↗4413.1 ± 947.4	→4828.7 ± 1182.9
	**MD -4**	→5025.3 ± 1197.2	→5154.4 ± 1184.4	→4998.9 ± 1212.8	→4145.6 ± 819.5	↗4712.9 ± 1167.6	→5060.4 ± 1286.4
	**MD -5**	→4344.5 ± 656.2	→4476.6 ± 684.2	→4349.9 ± 870.2	→3678.3 ± 884.7	→4187.6 ± 729.1	→4492.8 ± 675.9

**High speed (m)**	**Game**	**208.9 ± 90.54**	**267.2 ± 119.0**	**549.7 ± 186.2**	**228.9 ± 139.9**	**179.9 ± 79.2**	**668.4 ± 307.5**
	**MD -1**	↘111.9 ± 84.8	↘138.8 ± 82.6	↓151.7 ± 91.8	↓84.2 ± 69.1	↘126.5 ± 84.9	↓212.8 ± 129.1
	**MD -2**	↘107.9 ± 80.7	↓130.6 ± 106.2	↓236.4 ± 134.8	↘107.5 ± 71.3	↘107.9 ± 118.8	↓273.1 ± 161.2
	**MD -3**	↘146.7 ± 127.2	↘142.8 ± 105.1	↘293.2 ± 193.1	↘106.9 ± 85.6	→145.8 ± 141.0	↘338.8 ± 219.8
	**MD -4**	→166.9 ± 113.6	↘142.3 ± 94.9	↘298.9 ± 171.2	↘121.9 ± 92.2	→146.2 ± 100.1	↘313.4 ± 192.0
	**MD -5**	↓82.7 ± 99.0	↓61.1 ± 82.6	↓167.5 ± 218.6	↘59.1 ± 63.5	↘74.2 ± 89.0	↓181.2 ± 232.4

**RPE (AU)**	**Game**	**6.7 ± 1.73**	**6.6 ± 1.9**	**7.0 ± 1.5**	**5.7 ± 2.3**	**6.1 ± 1.3**	**7.4 ± 1.3**
	**MD -1**	↘5.3 ± 0.8	→5.6 ± 0.9	↘5.8 ± 0.7	→5.6 ± 0.9	→6 ± 1.0	↓5.5 ± 0.9
	**MD -2**	→6.4 ± 1.1	→6.9 ± 1.1	→6.2 ± 1.4	↗6.6 ± 0.8	→6.6 ± 0.9	↘6.6 ± 1.1
	**MD -3**	→6.6 ± 1.3	↗7.3 ± 0.8	→6.9 ± 1.3	↗7.0 ± 0.9	↗6.9 ± 0.8	→7.0 ± 1.1
	**MD -4**	→6.8 ± 1.1	→7.2 ± 1.0	→6.8 ± 1.4	↗6.8 ± 0.9	→6.8 ± 1.2	→7.1 ± 1.4
	**MD -5**	→6.5 ± 1.4	→7.1 ± 1.1	→7.3 ± 1.2	↗6.7 ± 1.0	↗7.0 ± 0.8	→6.9 ± 1.4

**RPE (AU)**	**Game**	**69.7 ± 15.9**	**74.1 ± 17.6**	**82.5 ± 21.9**	**56.3 ± 18.9**	**60.1 ± 10.1**	**89.9 ± 30.2**
	**MD -1**	↘54.8 ± 9.1	↘61.1 ± 9.3	↓54.6 ± 8.8	↘44.4 ± 9.2	↘53.8 ± 9.7	↓58.6 ± 11.1
	**MD -2**	→63.6 ± 12.7	→65.9 ± 10.6	↘62.2 ± 11.7	→49.4 ± 9.8	→58.1 ± 11.8	↘66.2 ± 13.7
	**MD -3**	→67.7 ± 13.2	→68.5 ± 9.1	↘69.9 ± 14.3	→54.1 ± 13.5	→63.8 ± 13.3	→75.7 ± 15.9
	**MD -4**	→70.5 ± 14.7	→73.1 ± 16.4	→78.9 ± 22.3	→55.5 ± 13.1	→67.9 ± 16.5	→80.4 ± 20.1
	**MD -5**	→63.2 ± 8.3	↘64.8 ± 9.7	→71.4 ± 14.4	→50.2 ± 13.9	→61.0 ± 10.9	↘72.9 ± 11.4

Note: MD: Match day; CB: Centre Backs; LB: Left Backs; LW: Left Wings ; PIV: Pivots; RB: Right Backs; RW: Right Wings; RPE: Rating of perceived exertion ; AU: Arbitray Units.

### Game demands

During games, right backs (RB) travelled (4187.6 ± 729.1 m) largely to very largely less TD (ES range from 1.8 to 2) than the other playing positions (left backs (LB): 4476.6 ± 684.2 m, RB: 4187.6 ± 729.1 m, CB: 4344.4 ± 656.1 m, LW: 4349.8 ± 870.2 m, RW: 4492.8 ± 675.8 m) except when they were compared to PIV (PIV: 3678.2 ± 884.6 m). Wing players (RW: 668.4 ± 307.5 m, LW: 549.6 ± 186.2 m) cover very largely more HSR distance (ES range from 2.3 to 3.3) during games when compared to the other playing positions (LB: 267.2 ± 119 m, PIV: 228.9 ± 139.9 m, CB: 208.92 ± 90.5 m, RB: 179.9 ± 79.2 m). During games, RB (60.1 ± 1 au) and PIV 56.3 ± 2 au) reported up to very largely less value (for RB, ES vs. LW = 2.3, vs. RW = 2.1, vs. LB and for RB vs. LW ES = 2.3, vs. RW = 2.03) than the other playing positions (range from 69.7 ± 15 au for CB to 90 ± 30.2 au for RW). They were no substantial differences in the RPE.

### Training sessions

#### Total distance

PIV travelled moderately to largely less distance (ES range from 1.2 to 1.6) in MD-5, MD-2, MD-1 (3678.3 ± 884.7 m, 3665.5 ± 597.6 m, 3526.4 ± 624.0 m) when compared to LB (4476.6 ± 684.2 m, 4731.6 ± 871.7 m, 4347.5 ± 623.6 m, respectively).

#### High-speed running

In MD-4, MD-3, and MD-2, the HSR distance travelled by PIV (MD-4: 392.8 ± 128.9 m, MD-3: 106.9 ± 85.6 m, MD-2: 107.5 ± 71.2 m) was largely to very largely lower (ES ranged from 1.3 to 2) than by wing players (MD-4, LW: 298.9 ± 171.2 m, RW: 313.4 ± 192 m, MD-3: LW: 293.2 ± 193.1 m, RW: 338.79 ± 219.8 m, MD-2: 236.4 ± 134.8 m, RW: 273.1 ± 161.2 m).

#### Player load

PIV showed the lowest PL value, up to largely less (ES ranged from 1.2 to 2.07) in all the training sessions and games (in MD-5: 50.2 ± 13.9 au, in MD-4: 55.5 ± 13.2 au, in MD-3: 54 ± 13.5 au, in MD-2: 49.4 ± 9.8 au, in MD-1: 44.4 ± 9.2 au). Wing players produced up to very largely higher PL (ES ranging from 1.5 to 1.8) demands in MD-5 (RW: 72.9 ± 11.4 au, LW: 71.4 ± 14.3 au), MD-4 (RW: 80.4 ± 20.1 au, LW: 78.8 ± 22.3 au), MD-3 (RW: 75.7 ± 15.9 au, LW: 69.9 ± 14.3 au).

LB showed the highest PL value in MD-1 (61.1 ± 9.3 au vs. 44.4 ± 9.1 au for PIV, LB vs. PIV, ES = 2.03).

#### Rating of perceived exertion

Like in matches, there were no substantial differences in the RPE, with values ranging from 5.72 ± 2.32 au for PIV to 7.43 ± 1.34 au for RW.

### Games vs. training

#### Within playing position differences

PIV showed moderate to large within-playing-position differences during games in TD (4951.1 ± 1047.6 m vs. 3218.4 ± 779.7 m, ES = 1.97), HSR (343.6 ± 84.2 m and 114.2 ± 70.3 m, ES = 1.91), PL (68.7 ± 15.5 au vs. 44 ± 13.2 au, ES = 1.77). There were moderate to very large differences (ES 1.44 to 2.06) for LB during games in TD (4161 ± 657.5 m vs. 5400.3 ± 980.5 m), in PL (87.2 ± 15.8 au vs. 75.3 ± 10.9 au) and RPE (7.7 ± 1 au vs. 5.6 ± 1.1 au). There were large to very large within-playing-position differences in HSR distance travelled by LB (306.2 ± 355.1 m vs. 83.1 ± 162.3 m).

PIV also showed some individual differences (ES = 1.84) in the PL in MD-5 (62.8 ± 13.8 au vs. 43.8 ± 9.2 au), MD-4 (63.0 ± 10.2 au vs. 51 ± 13 au), MD-3 (62.6 ± 10.9 vs. 44.2 ± 8.4 au), MD-2 (55.9 ± 9 au vs. 43.5 ± 6 au).

**FIG. 2:**
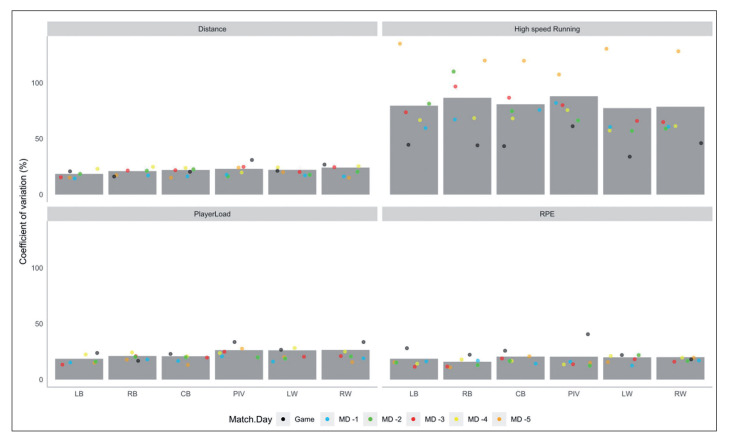
Coefficient of variation for all the metrics in each playing position for the different training days.

Figure 2 shows the coefficient of variation for all the metrics in each playing position for the different training days. HSR distance showed a higher variation (77.3% to 87.9%). When considering playing position and training days, HSR distance in MD-5 had the highest percentage of variation (107% for PIV to 135% for LB, 123.5 ± 9.8%). Games were the sessions with the lower CV (33.9% for LW to 61.1%, 45.5 ± 8.8%) for HSR.

**FIG. 3:**
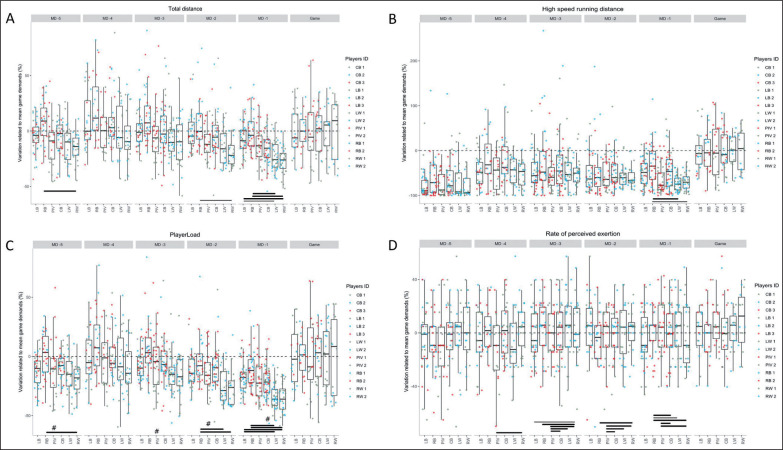
Comparison of mean percentage of game demands between playing position related to game day in total distance (A), high-speed running distance (B), player load (C), and rate of perceived exertion (D). Bar graphs represent the mean and standard deviation percentage of mean game demands for each playing position related to game day. Coloured dots stand for individual value within the playing position and game day. The thickness of the lines represents the magnitude of the difference (effect size): ━ very large difference; ─ large difference; ― moderate difference.

Figure 3 illustrates the difference between playing positions expressed in the percentage of variation related to the mean game demands in the different variables. LB and RB showed a moderately to largely higher percentage of mean game demands in MD-1 when compared to wing players in TD (LB: -7.5 ± 13.3%, RB: 0.41 ± 17.15%, LW: -22.6 ± 13.2%, RW: -26.8 ± 11.8%; ES from 1.14 to 1.81), in PL (LB: -17.4 ± 12.5%, RB: -10.5 ± 16%, LW: -33.8 ± 10.6%, RW: -34.9 ± 12.3% and ES from 1.38 to 1.71).

PIV showed up to largely higher percentage of mean game demands in RPE value in all training sessions (MD-5: 17.3 ± 17.6%, MD-4: 19.4 ± 16.1%, MD-3: 22.9 ± 16.8%, MD-2: 15.3 ± 14.2%, MD-1: -3 ± 15.5%, ES from 1.26 to 1.83) when compared to the other players (MD-1: from -15.6 ± 13.8% to -26.2 ± 12.5%, MD-2: range -11 ± 14% to 8.19 ± 13.9%, MD-3: 15 ± 13.3%, MD-4: -4.6 ± 18.5 to 11.4 ± 19.9%, MD-5: -3.3 ± 20% to 15.5 ± 12.6%).

RB followed the same pattern in MD-5, MD-3, MD-2, and MD-1 (ES from 1.14 to 1.68). RB (29.7 ± 47.17%) reached a moderately to large higher percentage of mean game demands in high-speed running compared to wing players (LW: -72.4 ± 16.7%, ES = 1.21, RW: -68.1 ± 19.3%, ES = 1.03) in MD-1.

There were large differences in the percentage of mean game demands between PIV in MD-2, MD-3, and MD-4 (respectively, -22.8 ± 10.7% vs. -0.8 ± 16.1%, ES = 1.6, -21.5 ± 14.9% vs. 11.1 ± 19.5%, ES = 1.85, -24.5 ± 5.3% vs 5.4 ± 23.5%, ES = 1.51).

## DISCUSSION

To our knowledge, this is the first time a handball team has been monitored longitudinally during a season in practices and games with a combination of IMUs and RPE. The most significant differences between playing positions were noted on match days, showing that training does not replicate game demands. PL and HSR were the metrics with the most important differences. When the difference between playing positions was expressed as the percentage of variation related to the mean game demands, the most important differences were found in MD-1 and the RPE.

### Match days

Regarding the external load in games, our results on match days are in line with previous studies. The wings (left (LW) and right (RW)) are, by far, the players who cover the most metres in HSR, both in training and in games. These results match those observed in earlier studies [5, 10, 12]. RW and LW were the players who covered the highest distance and RB the lowest. A similar effect has been found in previous studies [12].

Wings were also the players with the highest PL values compared to the other positions. These results differed from previous studies finding that CBs were the players with the highest PL value. Player rotation and team tactics could explain some of these differences because the nature of this playing time affects PL [31] substantially. In team handball, player rotations are unlimited and very easy to implement. As a result, many factors could explain this variation, such as players’ performance level (the better they are, the longer they tend to play), the tactical choice of the coach, or game model requirements. PL has been developed as a measure of physical performance based on changes in acceleration to capture non-running-based work (e.g., jumping, changes of direction, acceleration, contact) [5].

PIV and RB had the lowest IL concerning the other positions. This result could be related to different players’ rotation strategies. In many teams, RB are the players involved in the offensive/defensive systematic rotations for various reasons, such as a lack of defensive ability or fatigue management (RB need to be tall and left-handed and are essential for most teams). PIVs’ performances are paramount for many teams, and the physical demands they must cope with are high [9]. As a result, many coaches tend to establish more rotations for the PIVs.

### Training session

#### Total distance

PIV covered less distance than the other players during all training sessions (up to moderate to largely less than LB). The tactical and technical demands of the playing position likely play an important role. PIV players were a fixing point in the opponents’ defence when backs played more around the defensive system. A vital part of handball training is devoted to the stabilized phase (Table 2), accounting for most ball possession in matches [32]. This result should be related to the main objective, the relative part of the session’s static/full court phases, and the volume/intensity choice. It is expected that when training sessions are focused on static phases, PIVs do not accumulate many metres due to their defensive fixation function, performing more isometric or dynamic muscular actions. In turn, even if the training session is more dynamic (full-court situations), they tend to carry out the same function. Thus, they cover less distance and perform fewer running actions. These results are in line with those obtained in other studies where the PIV were the players who covered the lowest distance in official matches [5].

When expressed as a percentage of mean game demands, we can see that RB achieve a higher value than the other positions in many parts of training sessions. It is very likely that when there is a less stabilized phase in training (when coaches want their players to run more), the RB achieve a higher percentage as they run less than the other players in games. In training, all players tend to train simultaneously and perform the same tasks. These differences in RB (and to a lesser extent for LB) could be attributed to a higher rotation in playing time due to the demands on these positions according to the team’s playing model [5].

#### High-speed running distance

Wings covered higher HSR distance in all training sessions than the other playing positions. These results confirm that training requirements are not always in line with game demands [5, 10, 12]. HSR distance is an essential metric for performance [33] and injury prevention [34]. Our findings support the idea of Karcher & Buchheit [9] that there is a need for specific sprint training and hamstring prevention work for wings. The HSR percentage of mean game demands suggests that training sessions do not replicate the competition load as the HSR distance is lower than the match demands in all training sessions for all players (Figure 3). This lack of specificity can lead to some issues in some players, especially wings (who cover the highest HSR distance, such as decreasing their performance [5, 12] and increasing their risk of injury [9]. HSR distances should be close to or above those required by the competition to allow players to cope with these demands [17].

Moreover, the coefficient of variation of HSR showed that this variable fluctuated a lot. Large differences were found in all playing positions depending on MD. This variation suggests that the technical and tactical objectives of the training largely influence the HSR content and that this variable is not always adequately managed [9]. Our results indicate that HSR distance is mainly subjected to tactical choices.

#### Player load

Our results showed a large variation in PL when comparing the different playing positions. This fact confirms that each player needs a training load as individualized as possible; the external load demands differ for each playing position [5, 6, 29]. At the same time, it was observed that MD-4 was the day that was closest in terms of PL to the competitive needs. As seen in Figure 2, MD-4 was the day when the greatest volume of work was carried out both in terms of time and full court work and when the highest PL values could be accumulated.

PL is one of the most used variables to control the external load in training and competition in handball [5, 6, 29]. PIV were the players with the lower PL values in competition and the different training sessions. The specific demands of the position at a physical, technical, and tactical level may explain this result [5]. Most of the needs of these players are based on isometric strength work, as they were the players who were the most involved in contact actions and with few accelerations and lower high-velocity displacement [9, 22]. Wings showed the highest PL value in any training session, following the other external load variables (total distance and HSR). These differences between positions could be explained by the tactical demands of each position, their needs, and the game model used by the team [5].

#### Rate of perceived exertion

Our results suggest that players rated the session with similar intensities despite many differences in the external load (e.g., HSR distance). Surprisingly, the internal load did not reproduce the same pattern as the external loading. We could suggest two explanations for this offset. Firstly, the extensive experience of the players in hand-ball and years working with the same methodologies in the same club (3.3 ± 2.2 years in the club) may have caused a specific adaptation [17]. Secondly, many players’ characteristics influence internal load values, such as muscle mass, substrate concentrations [35], body size [22], or fitness level [36]. As a result, two players receive equivalent external loads, but the internal load could differ depending on individual characteristics. The player’s physical characteristics and body dimensions are position-dependent [6, 18, 21]. Overall, these factors mainly explain the results.

Comparing the values obtained during training sessions and games, we see that the results are often higher after training. These differences indicate that training and rest time density is much higher during the week than on MD. There are likely more in-game breaks due to the refereeing, the opponent’s game pace, and the player’s rotation strategies.

The differences in the percentage of variation considering the mean game demands are probably caused by the discrepancies between training and game values. Game demands fluctuate greatly when compared to the training context.

#### Coefficient of variation

The coefficient of variation is related to periodization, as variations in training load are a key and widely studied factor in performance [37]. Many studies suggest that poor training-load management and flawed prescription constitute significant risk factors for injury [38]. Our results show that games are the moment with the highest fluctuations in most of the variables. This is logical, considering that teams do not have control over the opponent’s executions. Most CVs are low and show a small magnitude (18 to 29%) except for HSR. HSR’s CV showed values ranging from 57 to 135%. Large differences were found in all playing positions depending on MD. This variation suggests that the technical and tactical objectives of training largely influence HSR content and that this variable is not managed [9]. These results indicate that HSR distance is a by-product of tactical choices. Too much variation in the same training days illustrates, from our point of view, that coaches do not control this variable. It is also worth noting that HSR distance was much higher during games than during training sessions, as training content does not replicate HSR game demands.

### Limitations

We could only study one professional team with a particular match and training model. The team’s profile (only one match per week) also allowed us to have stable microcycles that may not be generalizable to other contexts (teams playing more than one match per week, national teams). Another limitation is that the analysis of defensive specialists was not considered. Some players have a particular role, playing only during the defensive phase, and are not included in the attack. The type of training provided by the coaching staff, and its intensity, also influence the training demands to a large extent and can be a limitation. Other parameters, such as different playing strategies [39], the opponent’s level [40] and the use of a defensive specialist, may have revealed different results than ours.

### Practical applications

Coaches must consider variables such as positional demands or the team’s playing style to tailor training demands. Our results show that coaches should carefully evaluate several indicators to design the optimal training content. In our data, some values in the training sessions were above the game demands (total distance, RPE) and others under (HSR, PL). This scenario can be suboptimal to prepare players for the “worst case scenario” in competition.

It is worth noting that training demands differ largely between positions (wings and PIV vs. RB). Handball staff should use this information before designing microcycles to adjust training loads more precisely.

## CONCLUSIONS

We observed that internal load does not show the same pattern even if there are substantial differences in external load variables between playing positions. Our results confirm the need to control the load and the complementarity of the two types of load variables. One of the most relevant findings was the substantial variation in HSR between positions, games, and training sessions. Coaches must give special attention to HSR because it is an essential variable for injury prevention (9) and performance (12).

Further research is needed in handball to study the parameters that influences the behaviour of internal and external loading variables to optimize training.
